# Factors associated with health-related quality of life in adults with asthma in Jordan: the roles of asthma control and asthma-related numeracy

**DOI:** 10.1080/21642850.2026.2760023

**Published:** 2026-11-07

**Authors:** Walid Al-Qerem, Anan Jarab, Khaled Al Oweidat, Majd Kasasbeh, Lujain Al-sa’di, Judith Eberhardt

**Affiliations:** a Department of Pharmacy, Faculty of Pharmacy, Al-Zaytoonah University of Jordan, Amman, Jordan; b Department of Clinical Pharmacy, Faculty of Pharmacy, Jordan University of Science and Technology, Irbid, Jordan; c Pulmonary and Sleep Department, The University of Jordan, Amman, Jordan; d Department of Psychology, School of Social Sciences, Humanities and Law, Teesside University, Middlesbrough, United Kingdom

**Keywords:** Asthma, health-related quality of life, asthma control, asthma-related numeracy, medication adherence

## Abstract

**Background:**

Health-related quality of life (HRQoL) is an important outcome in asthma care. Evidence from Middle Eastern settings remains limited regarding the relative contribution of clinical, treatment-related, sociodemographic, and self-management factors to generic HRQoL.

**Objective:**

To identify factors independently associated with HRQoL among adults with asthma in Jordan, focusing on asthma-related numeracy and asthma control, and to test the robustness of findings in a sensitivity analysis.

**Methods:**

This cross-sectional study included 396 adults with asthma. The EQ−5D−3L index was modelled using hierarchical ordinary least squares regression with HC3 robust standard errors. Predictors included sociodemographic characteristics, medication use, Ar-ANQ score, medication adherence, and asthma-control status. A sensitivity model used a three-level treatment-intensity and adherence-based clinical-status proxy.

**Results:**

Partly controlled and uncontrolled asthma were associated with lower EQ−5D−3L index values than well-controlled asthma; the adjusted difference for uncontrolled asthma was −0.225 (95% CI −0.271 to −0.179). Each one-point higher Ar-ANQ score was associated with a 0.024-point higher index (95% CI 0.005 to 0.043). Older age and oral corticosteroid use were also associated with lower HRQoL, whereas high adherence was not independently associated with the outcome. In the sensitivity analysis, both difficult-to-treat and severe-asthma proxy groups had lower HRQoL than the non-severe group.

**Conclusion:**

Asthma control was the strongest independent correlate of generic HRQoL. Asthma-related numeracy showed a smaller positive association, while adherence was not independently associated after adjustment. These findings support a multifactorial interpretation of asthma HRQoL and do not imply causality.

## Introduction

Chronic diseases, such as asthma and other respiratory conditions, pose significant global health burdens, contributing to reduced quality of life, frequent healthcare utilization, and premature mortality (Oh et al., 2025; Soriano et al., 2020). Effective disease management in these conditions requires more than pharmacological treatment alone; it also relies on patient-centered factors such as medication adherence, health literacy, and disease self-management behaviors (Bourbeau & Van Der Palen, 2009).

Asthma, in particular, remains one of the most prevalent chronic respiratory diseases worldwide, affecting over 262 million people and causing nearly 461,000 deaths annually, according to the World Health Organization (Asthma, n.d). In adults, asthma significantly impairs daily functioning, reduces productivity, and increases the risk of exacerbation and hospitalization. Asthma prevalence in the Middle East varies across studies and countries, with reported rates ranging from approximately 4.4% to 15% among adults. For instance, the SNAPSHOT study observed adjusted asthma prevalence rates of 6.7% in Turkey, 7.6% in Egypt, and 4.4% in the Gulf Cluster countries, including Kuwait, Saudi Arabia, and the United Arab Emirates (Tarraf et al., 2018). The chronic burden of asthma in these regions underscores the need for enhanced management strategies that address both clinical and behavioral determinants of disease control and quality of life.

Health-related quality of life (HRQoL), including preference-based measures such as the EQ−5D, is a central outcome in clinical and health-services research because it reflects perceived functioning and well-being (Rabin & Charro, 2001). Generic HRQoL instruments permit comparison across diseases and populations (Tan et al., 2013; Wei et al., 2021). In asthma, worsening control is consistently associated with lower generic and disease-specific HRQoL (Chen et al., 2007). However, the relative associations of sociodemographic, behavioral, treatment-related, and clinical factors with HRQoL remain insufficiently characterized in middle-income settings.

Health literacy has emerged as a crucial modifiable determinant of adherence and disease outcomes. Defined as the degree to which individuals can obtain, process, and understand basic health information and services needed to make appropriate health decisions (Nutbeam, 2008), health literacy is closely connected to educational and socioeconomic status and health outcomes (Berete et al., 2024). Low health literacy has been linked to poor medication adherence, inadequate disease knowledge, and lower HRQoL across various chronic illnesses (Berkman et al., 2011; McCormack et al., 2017).

Asthma-related numeracy is a narrower functional domain within health literacy and concerns the ability to interpret probabilities, medication doses, and peak-flow information. Previous asthma studies have linked lower numeracy and lower health literacy with poorer asthma-related quality of life and other outcomes, although the magnitude and independence of these relationships have varied after adjustment for socioeconomic and clinical factors (Apter et al., 2006, 2009).

Few studies in Middle Eastern populations have examined asthma-related numeracy, medication adherence, and asthma control together in relation to generic HRQoL. Their additional explanatory contribution beyond sociodemographic characteristics and medication use remains unclear.

The primary objective of this study was to identify factors independently associated with the EQ−5D−3L index among adults with asthma in Jordan. Secondary objectives were to quantify the incremental explanatory contributions of asthma-related numeracy, medication adherence, and asthma-control status using hierarchical regression and to assess the robustness of the findings in a sensitivity model incorporating a treatment-intensity and adherence-based clinical-status proxy.

## Materials and methods

This cross-sectional study included adult patients with asthma who were attending the pulmonary outpatient clinic at Jordan University Hospital, located in Amman, Jordan. Data were collected between November 2024 and April 2025. Eligibility criteria included individuals aged 18 years or older with a clinical diagnosis of asthma for at least one year, and who were willing to participate. Participants were recruited using consecutive non-probability sampling. Clinical records of patients with scheduled clinic appointments were reviewed in advance to assess eligibility. All eligible patients attending the clinic during the recruitment period were approached consecutively while waiting for their appointments and invited to participate. Before participation, individuals were informed of the study’s objectives and assured of data confidentiality and their right to withdraw at any time. Written informed consent was obtained from each participant. The self-administered questionnaire required approximately 15 minutes to complete. The questionnaire was provided in an electronic format that required all items to be completed before submission, which prevented missing data. The study adhered to the ethical principles of the Declaration of Helsinki. Ethical approval was obtained from both Al-Zaytoonah University of Jordan (Reference No. 47/14/2023-2024) and the University of Jordan Hospital (Reference No. 10/2024/15816).

### Data collection and survey instruments

The study instrument included a sociodemographic sheet covering age, sex, educational level, and monthly household income. Lower education was defined as high school or less, and higher education as a college or university degree. Lower and higher monthly household income were defined as < 500 and ≥ 500 Jordanian dinars (JOD), respectively. It also included the Arabic version of the Asthma Numeracy Questionnaire (Ar-ANQ) (Al-Qerem et al., 2025), a validated four-item measure of asthma-related numeracy, which is a functional component of health literacy. The first item assessed calculation of an oral corticosteroid dose, the second assessed interpretation of osteoporosis risk, and the third and fourth items involved peak-flow calculations. Each correct answer received one point, yielding a total score from 0 to 4.

Additionally, the study instrument included the Arabic version of the Medication Adherence Report Scale (MARS−5) (Al-Qerem et al., 2022), which evaluated medication adherence by assessing whether participants forgot, altered, discontinued, or skipped doses. The questionnaire comprised five Likert-scale items scored from 1 (‘Always’) to 5 (‘Never’), yielding a total score of 5–25, with higher scores indicating better adherence. The adherence scores were dichotomized into high adherence (MARS−5 ≥ 24) and low adherence (MARS−5 ≤ 23) (coded as 0 = low adherence, 1 = high adherence). This cut-off was selected a priori based on recent MARS−5 research, in which thresholds in the 23–25 range have been used to define high adherence, and this choice has often been justified by ROC analyses against external adherence criteria (Jožef et al., 2024; Norberg et al., 2022; Spetz et al., 2024; van de Steeg et al., 2024). The dichotomization was used to provide a clinically interpretable adherence status while remaining consistent with the existing literature on MARS−5.

Asthma symptom control was assessed using the four-item GINA symptom-control assessment, referred to in this study as ASC (GINA-2024-Strategy-Report-24_05_22_WMS, n.d). The four yes/no items assessed the frequency of daytime symptoms, night waking due to asthma, reliever medication use, and activity limitation due to asthma over the preceding four weeks. Responses were scored as 0 for “No” and 1 for “Yes.” Participants with no positive responses were classified as well controlled, those with one or two positive responses as partly controlled, and those with three or four positive responses as uncontrolled. The study also included the EQ−5D−3L (EuroQol, 1990), a preference-based descriptive system covering mobility, self-care, usual activities, pain/discomfort, and anxiety/depression, with three response levels for each dimension. Participants completed the EQ−5D−3L, and utility indices were calculated from their three-level health-state profiles using the EuroQol-provided SPSS reverse-crosswalk syntax for EQ−5D−3L. This syntax is specifically intended to score EQ−5D−3L responses, with utility values derived from the England EQ−5D−5L value set (Devlin et al., 2018; EuroQol Research Foundation, 2020). Higher index values indicate better HRQoL.

In the sensitivity analysis, asthma control status was not entered as a separate predictor and was regarded as a measure of current symptom control rather than formal disease severity. Clinical status was defined using an ERS/ATS-aligned treatment-requirement criterion, comprising asthma requiring oral corticosteroids or high-dose inhaled corticosteroid/long-acting β₂-agonist therapy to maintain control, or asthma remaining uncontrolled despite high-dose inhaled corticosteroid/long-acting β₂-agonist and/or oral corticosteroid therapy (Chung et al., 2014). Consequently, participants could meet the treatment-based severity criterion despite being currently controlled when control depended on high-intensity therapy. Participants who did not meet this criterion were classified as non-severe. Among those who met it, the binary adherence classification was used to distinguish between the difficult-to-treat proxy group, defined by low adherence, and the severe-asthma proxy group, defined by high adherence. These classifications were treated as research proxies rather than confirmed clinical diagnoses because information on inhaler technique, treatment optimization, contributory factors, and longitudinal response to therapy was unavailable.

### Instrument validation

The Arabic Asthma Numeracy Questionnaire (Ar-ANQ) was validated in a previously published study using the same cohort (Al-Qerem et al., 2025). Confirmatory factor analysis (CFA) and Rasch modelling supported its unidimensional structure and reliability. The published item-level CFA findings are summarized in Supplementary Table S1.

For the present study, CFAs of the EQ−5D−3L, MARS−5, and asthma symptom-control items were conducted using diagonally weighted least squares (DWLS), with polychoric or tetrachoric correlations as appropriate for ordinal or binary indicators. The EQ‑5D‑3L items (mobility, self-care, usual activities, pain/discomfort, and anxiety/depression) were adequately represented by a single factor, with strong overall fit (CFI = 0.997, TLI = 0.994, RMSEA = 0.086, SRMR = 0.054) and high standardized factor loadings. Internal consistency was supported by McDonald’s omega and polychoric alpha coefficients. Similarly, the MARS‑5 items supported a one-factor structure with excellent global fit (CFI = 1.00, RMSEA = 0.00, SRMR = 0.03) and acceptable standardized loadings, with reliability indices indicating adequate internal consistency. The asthma control instrument was also modelled as a single factor; although RMSEA and SRMR were comparatively inflated, this pattern can occur in models with few binary indicators and low degrees of freedom and was interpreted in conjunction with the remaining fit indices and the magnitude of factor loadings. Full item-level CFA outputs for EQ‑5D‑3L, MARS‑5, and the asthma control instrument, including standardized loadings, SEs, item R^2^ values, and global fit indices, are provided in the Supplementary Materials (Tables S2–S4).

### Analytic sample size

An a-priori sample-size calculation was performed for the planned multivariable analysis using a fixed-model multiple linear regression framework. Based on the more highly parameterized primary model, with 14 slope coefficients, an anticipated small-to-moderate effect size (f^2^ = 0.05), a significance level of 0.05, and 80% statistical power, the minimum required sample was 379 participants. The regression analytic sample comprised 396 participants.

### Statistical analysis

All analyses were conducted in R version 4.5.3. The EQ−5D−3L index was the dependent variable. Continuous variables are summarized using means and standard deviations, and categorical variables using frequencies and percentages. The primary analysis used hierarchical ordinary least squares regression. Model 1 included age, sex, education, income, and six medication-use indicators (montelukast, inhaled corticosteroids, antihistamines, oral corticosteroids, ipratropium bromide, and antibiotics). Model 2 added Ar-ANQ score, Model 3 added binary adherence status, and Model 4 added asthma-control category. Both adjusted unstandardized regression coefficients (B), with 95% confidence intervals, and standardized regression coefficients (*β*) are reported. Standardized coefficients were calculated as *β* = B × SD(X)/SD(Y) using the final model-matrix columns; therefore, *β* values for categorical indicators reflect their observed sample prevalence. Age was scaled per 10-year increase; sex, education, income, medication use, and adherence were entered as categorical variables; Ar-ANQ score was continuous; and asthma control was modeled as a nominal three-level factor with well-controlled asthma as the reference category.

Because the Breusch-Pagan test indicated heteroskedasticity, HC3 heteroskedasticity-robust standard errors and *p*-values were used for inference. Model performance was summarized using R^2^ and adjusted R^2^. The incremental contribution of each predictor block was quantified using the change in unadjusted R^2^ (ΔR^2^) between successive nested models. Multicollinearity was assessed using variance inflation factors, and observations exceeding Cook's distance of 4/N were identified for influence review.

The sensitivity analysis replaced medication indicators, adherence, and asthma-control category with the three-level adherence-refined clinical-status proxy. This model retained age, sex, education, income, and Ar-ANQ. The non-severe group was the reference category.

Standardized coefficients (*β*) were reported for all predictors directly estimated in the regression models. In the sensitivity model, non-severe asthma was the reference category; therefore, the model directly estimated the difficult-to-treat-versus-non-severe and severe-versus-non-severe comparisons. The additional difficult-to-treat-versus-severe comparison was calculated from the same fitted model as a linear contrast of these two coefficients. Because this contrast was not a separate coefficient under the selected reference coding, it is reported as an HC3-robust adjusted unstandardized mean difference (B), with its 95% confidence interval and *p* value, without a standardized *β*. Statistical significance was evaluated using two-sided *p* values at *α* = .05.

## Results

The analytic sample comprised 396 adults with asthma. Mean age was 50.87 years (SD = 15.90); 216 participants (54.5%) were female, 212 (53.5%) held a college/university degree, and 302 (76.3%) had a monthly household income below 500 JOD (Table 1). Among the medication classes included in the analysis, inhaled corticosteroids were reported by 306 participants (77.3%), antihistamines by 155 (39.1%), montelukast by 88 (22.2%), oral corticosteroids by 86 (21.7%), antibiotics by 65 (16.4%), and ipratropium bromide by 61 (15.4%). Medication categories were not mutually exclusive (Table S5). Mean Ar-ANQ score was 1.78 (SD = 1.54), and mean EQ−5D−3L index was 0.825 (SD = 0.176). Low adherence was recorded for 215 participants (54.3%) and high adherence for 181 (45.7%). Asthma was well controlled in 141 participants (35.6%), partly controlled in 176 (44.4%), and uncontrolled in 79 (19.9%).

**Table 1.** Characteristics of the analytic sample (*N* = 396).

Variable	Mean (SD) or *n* (%)
Age, years	50.87 (15.90)
Sex: female	216 (54.5%)
Sex: male	180 (45.5%)
Education: high school or less	184 (46.5%)
Education: college/university degree	212 (53.5%)
Monthly household income: < 500 JOD	302 (76.3%)
Monthly household income: ≥ 500 JOD	94 (23.7%)
Ar-ANQ score	1.78 (1.54)
EQ−5D−3L index	0.825 (0.176)
Adherence: low	215 (54.3%)
Adherence: high	181 (45.7%)
Asthma control: well controlled	141 (35.6%)
Asthma control: partly controlled	176 (44.4%)
Asthma control: uncontrolled	79 (19.9%)
Clinical status: non-severe	259 (65.4%)
Clinical status: difficult-to-treat proxy	100 (25.3%)
Clinical status: severe-asthma proxy	37 (9.3%)

Note: The treatment criterion was requiring oral corticosteroids or high-dose ICS/LABA to maintain control, or remaining uncontrolled despite these treatments. Non-severe: criterion not met; difficult-to-treat proxy: criterion met with low adherence (MARS−5 ≤ 23); severe-asthma proxy: criterion met with high adherence (MARS−5 ≥ 24).

In the fully adjusted primary model, Ar-ANQ score remained positively associated with EQ−5D−3L index (B = 0.024, *β* = 0.206, 95% CI 0.005 to 0.043; *p* = .015). Relative to well-controlled asthma, partly controlled asthma was associated with an adjusted index 0.065 points lower (*β* = −0.184, 95% CI −0.092 to −0.039; *p* < .001), and uncontrolled asthma was associated with an index 0.225 points lower (*β* = −0.512, 95% CI −0.271 to −0.179; *p* < .001) (Table 2). Older age was independently associated with lower EQ−5D−3L index (B per year = −0.0022, *β* = −0.197, 95% CI −0.0034 to −0.0009; *p* < .001), equivalent to B = −0.022 per 10 years as reported in Table 2. Oral corticosteroid use was also associated with lower EQ−5D−3L index (B = −0.041, *β* = −0.097, 95% CI −0.076 to −0.007; *p* = .018). High adherence was not independently associated with the index after adjustment (B = −0.026, *β* = −0.074, 95% CI −0.069 to 0.017; *p* = .230). The remaining demographic and medication coefficients were not statistically significant.

**Table 2.** Primary multivariable linear regression of factors associated with EQ−5D−3L index (*N* = 396).

Predictor	B	β	Robust SE	95% CI	*p*
Age, per 10 years	−0.022	−0.197	0.006	[−0.034, −0.009]	< .001
Male sex	0.005	0.015	0.014	[−0.023, 0.034]	.710
College/university degree	−0.006	−0.017	0.016	[−0.038, 0.026]	.712
Monthly household income ≥ 500 JOD	0.016	0.038	0.015	[−0.013, 0.045]	.288
Montelukast use	0.027	0.063	0.017	[−0.007, 0.060]	.123
ICS use	−0.023	−0.056	0.013	[−0.050, 0.003]	.081
Antihistamine use	−0.021	−0.059	0.015	[−0.050, 0.008]	.154
Oral corticosteroid use	−0.041	−0.097	0.017	[−0.076, −0.007]	.018
Ipratropium bromide use	0.007	0.015	0.022	[−0.035, 0.050]	.732
Antibiotic use	−0.032	−0.068	0.024	[−0.080, 0.015]	.185
Ar-ANQ score, per point	0.024	0.206	0.010	[0.005, 0.043]	.015
High adherence	−0.026	−0.074	0.022	[−0.069, 0.017]	.230
Partly controlled asthma	−0.065	−0.184	0.014	[−0.092, −0.039]	< .001
Uncontrolled asthma	−0.225	−0.512	0.023	[−0.271, −0.179]	< .001

Note: B, adjusted unstandardized regression coefficient; *β*, standardized regression coefficient. HC3 robust SEs, 95% CIs, and *p* values refer to B. For categorical predictors, *β* reflects observed category prevalence. Reference categories were female sex, high school or less, monthly household income < 500 JOD, no medication use, low adherence, and well-controlled asthma. Age was scaled per 10-year increase. Ar-ANQ, Arabic Asthma Numeracy Questionnaire; CI, confidence interval; ICS, inhaled corticosteroids; SE, standard error.

Model R² = 0.478; adjusted R² = 0.459. Breusch-Pagan *p* < .001; maximum adjusted GVIF = 1.52.

The initial model containing demographics and medication-use indicators explained 25.6% of the variance in EQ−5D−3L index (R^2^ = 0.256; adjusted R^2^ = 0.237). Adding Ar-ANQ score increased R^2^ by 0.040 (adjusted R^2^ = 0.276), adherence increased R^2^ by 0.007 (adjusted R^2^ = 0.282), and asthma control increased R^2^ by 0.174 (adjusted R^2^ = 0.459) (Table 3). The maximum adjusted GVIF was 1.52. HC3 inference was used because the Breusch-Pagan test was significant (*p* < .001). Figure 1 summarizes the selected adjusted associations from the primary and sensitivity models. The full coefficient estimates are reported in Tables 2 and 4.

**Table 3.** Hierarchical model performance for EQ−5D−3L index (*N* = 396).

Model	Predictor block	Adjusted R^2^	ΔR^2^
Model 1	Demographics+medication indicators	0.237	0.256
Model 2	Model 1+Ar-ANQ	0.276	0.040
Model 3	Model 2+binary adherence	0.282	0.007
Model 4	Model 3+asthma-control category	0.459	0.174

Note: ΔR^2^ is calculated from unadjusted R^2^ and is relative to the preceding model; Model 1 is shown relative to the intercept-only model. Ar-ANQ, Arabic Asthma Numeracy Questionnaire.

**Figure 1.**
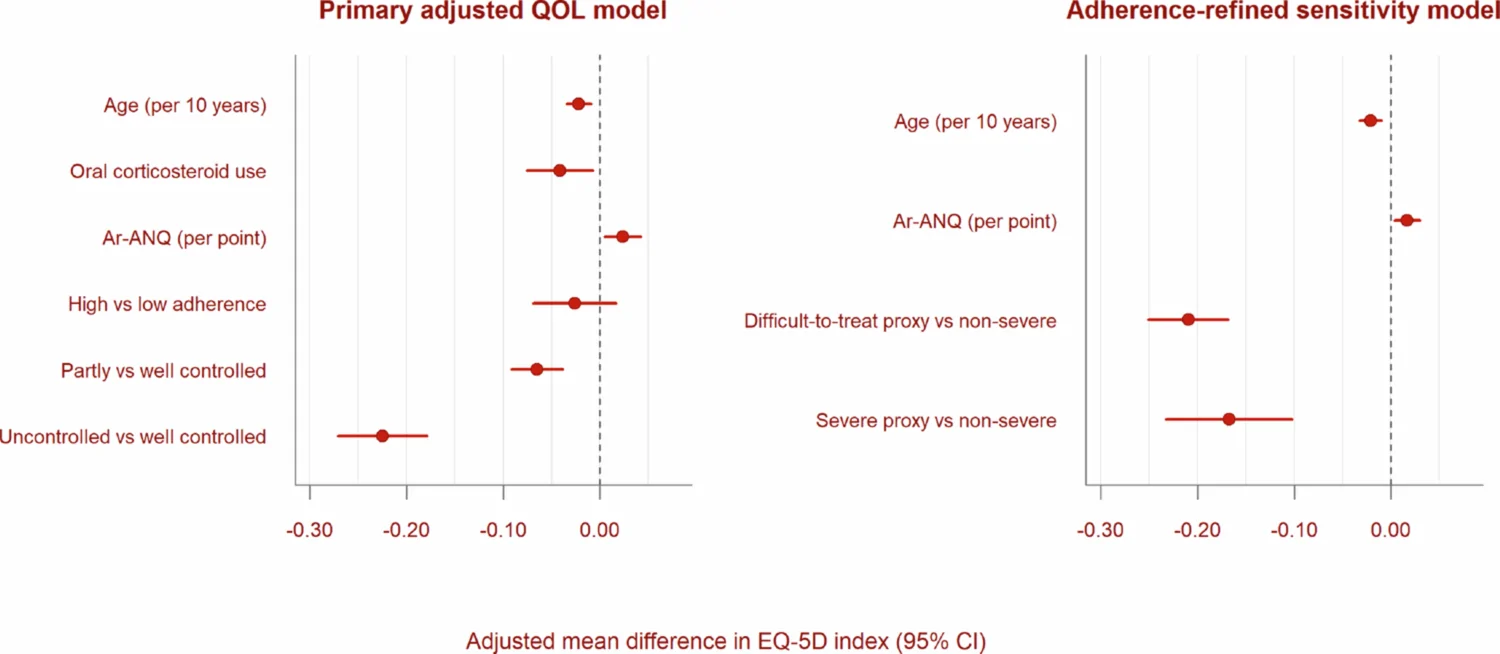
Selected adjusted associations with EQ−5D−3L index in the primary and adherence-refined sensitivity models. Points show unstandardized adjusted mean differences and horizontal lines show HC3 95% confidence intervals. The vertical dashed line indicates no association. Ar-ANQ, Arabic Asthma Numeracy Questionnaire; CI, confidence interval.

In the adherence-refined sensitivity model, 259 participants were classified as non-severe, 100 as difficult-to-treat proxies based on a treatment-based severity flag plus low adherence, and 37 as severe-asthma proxies based on the same treatment-based flag plus high adherence. Relative to the non-severe group, adjusted EQ−5D−3L index was lower in the difficult-to-treat proxy group (B = −0.210, *β* = −0.519, 95% CI −0.251 to −0.168; *p* < .001) and the severe-asthma proxy group (B = −0.167, *β* = −0.278, 95% CI −0.232 to −0.102; *p* < .001) (Table 4). The adjusted difference between the difficult-to-treat and severe-asthma proxy coefficients was not statistically significant (B = −0.042, 95% CI −0.121 to 0.037; *p* = .296). Ar-ANQ score remained positively associated with EQ−5D−3L index (B = 0.017, *β* = 0.147, 95% CI 0.004 to 0.030; *p* = .013). The sensitivity model had an adjusted R^2^ of 0.470.

**Table 4.** Adherence-refined clinical-status sensitivity model for EQ−5D−3L index (*N* = 396).

Predictor or contrast	B	β	Robust SE	95% CI	*p*
Age, per 10 years	−0.021	−0.189	0.006	[−0.032, −0.009]	<.001
Male sex	0.001	0.004	0.014	[−0.026, 0.029]	.925
College/university degree	−0.018	−0.052	0.015	[−0.048, 0.011]	.226
Monthly household income ≥ 500 JOD	0.011	0.026	0.015	[−0.019, 0.040]	.482
Ar-ANQ score, per point	0.017	0.147	0.007	[0.004, 0.030]	.013
Difficult-to-treat proxy	−0.210	−0.519	0.021	[−0.251, −0.168]	<.001
Severe-asthma proxy	−0.167	−0.278	0.033	[−0.232, −0.102]	<.001
Difficult-to-treat minus severe proxy	−0.042	N/A	0.040	[−0.121, 0.037]	.296

Note: B, adjusted unstandardized coefficient; *β*, standardized coefficient. HC3 robust SEs, 95% CIs, and *p* values refer to B. For categorical predictors, *β* reflects observed category prevalence. *β* is not applicable to the post-estimation difficult-to-treat-minus-severe contrast. The severity-candidate flag plus low or high binary adherence defined the difficult-to-treat and severe-asthma proxies, respectively; medication indicators, adherence, and control were otherwise excluded. These are not confirmed diagnoses because inhaler technique, treatment optimization, and longitudinal response were unavailable. References: female sex, high school or less, monthly household income < 500 JOD, and non-severe asthma. Model R^2^ = 0.479; adjusted R^2^ = 0.470. Ar-ANQ, Arabic Asthma Numeracy Questionnaire; CI, confidence interval; SE, standard error.

The sensitivity model showed no evidence of problematic multicollinearity (maximum adjusted GVIF = 1.33). Because clinical optimization and inhaler-technique data were unavailable, the difficult-to-treat and severe categories are interpreted as research proxies rather than confirmed clinical diagnoses.

## Discussion

This study evaluated a broad set of factors associated with generic HRQoL among adults with asthma in Jordan. The results favor a multifactorial interpretation rather than a health-literacy-centered account. Asthma-control status accounted for the largest incremental increase in R^2^ and showed the largest coefficients, whereas asthma-related numeracy retained a smaller positive association. Older age and oral corticosteroid use were also associated with lower EQ−5D−3L index values, while binary adherence was not independently associated with the outcome after simultaneous adjustment.

The mean EQ−5D−3L index of 0.825 was slightly higher than the pooled utility estimate of approximately 0.78 reported in a systematic review of adult asthma studies. Such comparisons should remain cautious because utility values depend on the national value set, clinical case mix, and the distributions of symptom control and disease severity. Nevertheless, the present mean falls within the range reported across adult asthma populations and indicates relatively preserved average generic health status despite substantial heterogeneity in control (Oh et al., 2022).

Asthma control was the clearest correlate of HRQoL. Compared with well-controlled asthma, partly controlled and uncontrolled asthma were associated with adjusted EQ−5D−3L decrements of 0.065 and 0.225, respectively. The direction and graded pattern agree with pooled evidence showing progressive utility loss across well-controlled, partly controlled, and uncontrolled states, and with prospective evidence that poor control predicts subsequent impairment in both disease-specific quality of life and EQ−5D utility independently of baseline severity (Chen et al., 2007; Oh et al., 2022). Earlier cross-sectional work in Spain and Jordan similarly identified poor control as one of the most consistent factors associated with reduced HRQoL (Gonzalez-Barcala et al., 2012; Jarab et al., 2023). Although the adjusted decrement was greater for uncontrolled than partly controlled asthma, no explicit benchmark was applied to assess the clinical importance of these differences. These cross-sectional associations do not demonstrate that treatment or improved asthma control would produce corresponding gains in HRQoL.

Asthma-related numeracy remained positively associated with EQ−5D−3L after adjustment: each one-point higher Ar-ANQ score was associated with a 0.024-point higher index (95% CI 0.005 to 0.043). The clinical importance of this association remains uncertain in the absence of an explicit benchmark. Although previous studies assessed asthma-specific rather than generic HRQoL, the direction of this finding is consistent with earlier research showing that higher asthma-related numeracy was associated with better asthma-related quality of life, including after adjustment for demographic characteristics (Apter et al., 2009, 2013). The Ar-ANQ, however, assesses a focused set of numerical tasks involving oral corticosteroid dosing, probability, and peak-flow interpretation rather than the broader multidimensional construct of health literacy (Al-Qerem et al., 2025; Nutbeam, 2008). The observed association may therefore reflect a greater ability to interpret numerical information required for asthma self-management. However, it may also partly capture underlying socioeconomic differences or greater self-efficacy, as previous research found that household income and, to a lesser extent, self-efficacy attenuated the association between asthma numeracy and asthma-related quality of life (Apter et al., 2009).

Older age was independently associated with lower EQ−5D−3L, which is consistent with previous studies in which advancing age was related to poorer asthma HRQoL (Gonzalez-Barcala et al., 2012; Jarab et al., 2023). Oral corticosteroid use was also independently associated with lower EQ−5D. This association could be indicative of greater asthma severity or a higher disease and exacerbation burden, as patients with more severe or difficult-to-control asthma may be more likely to require systemic corticosteroid therapy. In addition, it may reflect corticosteroid-related adverse effects, which could further impair HRQoL. Because information on treatment indication, dose, duration, cumulative exposure, and exacerbation history was unavailable, the relative contributions of disease severity and medication-related adverse effects could not be determined (Persaud et al., 2023).

The absence of an independent adherence association requires a restrained interpretation. The fully adjusted coefficient addresses whether adherence is associated with HRQoL independently of asthma control; it does not test whether adherence may be related to HRQoL through control. Self-reported adherence is also vulnerable to recall and social-desirability error, and dichotomization at a high threshold reduces information. Intervention evidence indicates that digital strategies can improve adherence and, in some settings, asthma control, whereas effects on quality of life are smaller and less consistent (Apter et al., 2013; Chan et al., 2022).

The sensitivity analysis produced a clinically coherent pattern: both treatment-intensive proxy groups had substantially lower EQ−5D−3L index values than the non-severe group, and the difference between the two proxy groups was imprecise and not statistically significant. Severe asthma is known to impose a marked quality-of-life burden (Hossny et al., 2017). However, GINA definitions require confirmation of the diagnosis, optimized high-dose treatment, good adherence, correct inhaler technique, and management of contributory factors before asthma is classified as severe (Global Initiative for Asthma, 2026). Because these elements were not fully observed, the categories in this study should remain explicitly labeled as research proxies. The non-significant contrast does not establish equivalence, particularly given the small severe-asthma proxy group.

### Study recommendations, strengths, and limitations

The strongest clinical implication is the need to assess asthma control and generic HRQoL systematically, particularly among patients with uncontrolled symptoms or treatment-intensive profiles. A comprehensive review should include diagnostic confirmation, inhaler technique, adherence barriers, comorbidities, environmental exposures, exacerbation history, and corticosteroid burden rather than reliance on any single behavioral indicator (Global Initiative for Asthma, 2026).

Asthma-related numeracy may still provide useful information for tailoring communication. Patients who have difficulty interpreting doses, probabilities, or peak-flow values may benefit from simpler numerical explanations, visual formats, teach-back, and written action plans. These strategies are plausible and low risk, but the current cross-sectional association does not demonstrate that improving numeracy will increase EQ−5D−3L utility.

Methodological strengths include a prespecified hierarchical sequence, treatment of asthma control as a nominal variable, HC3 robust inference in the presence of heteroskedasticity, reporting of incremental R^2^ change (ΔR^2^), and a sensitivity analysis that did not re-enter variables used to construct the clinical-status proxy. The analysis also distinguished current symptom control from treatment-requirement-based severity, reducing conceptual conflation between related but non-identical constructs.

Several limitations affect interpretation. The cross-sectional design precludes temporal and causal inference. Adherence, asthma control, and HRQoL were self-reported and may be affected by recall, social-desirability, and common-method bias. The Ar-ANQ is a four-item measure of asthma-related numeracy rather than a comprehensive health-literacy instrument. Residual confounding is likely because smoking, comorbidities, exacerbation frequency, inhaler technique, and cumulative oral corticosteroid exposure were unavailable. The single-center sample may limit generalizability. In addition, the generic EQ−5D−3L does not provide the symptom-specific detail available from asthma-specific quality-of-life instruments.

Longitudinal and interventional studies should examine whether asthma-related numeracy predicts subsequent change in control or HRQoL and whether numeracy-sensitive communication improves patient outcomes. Future analyses should use objective adherence measures, verified medication intensity and duration, exacerbation history, comorbidity profiles, inhaler-technique assessment, and formal severe-asthma adjudication after treatment optimization. Larger samples are needed to compare difficult-to-treat and severe asthma with adequate precision.

## Conclusion

In this cross-sectional study, asthma-control status was the dominant independent correlate of EQ−5D−3L, while asthma-related numeracy showed a smaller positive association and binary adherence was not independently associated with the outcome. Older age and oral corticosteroid use were also associated with lower generic HRQoL. The sensitivity analysis identified substantial HRQoL deficits in both treatment-intensive proxy groups. Taken together, the findings indicate that HRQoL in this population was most closely associated with current asthma control, while asthma-related numeracy contributed additional but more limited explanatory information. The results do not establish causal effects of numeracy, adherence, control, or treatment.

## Supplementary Material

Supplementary_Clean.docx Supplementary_Clean.docx

## Data Availability

The data that support the findings of this study are openly available in Zenodo at https://doi.org/10.5281/zenodo.15166616, reference number 15166616.

## Open scholarship

This article has earned the Center for Open Science badge for Open Data. The data and materials are openly accessible at https://doi.org/10.5281/zenodo.15166616. To obtain the author's disclosure form, please contact the Editor.
