# Supplementary material for: Factors associated with health-related quality of life in adults with asthma in Jordan: the roles of asthma control and asthma-related numeracy
Source: Health Psychol Behav Med. 2026 Nov 7;14(1):2760023. doi: 10.1080/21642850.2026.2760023 (PMC13757899; doi:10.1080/21642850.2026.2760023)
Supplement: Supplementary file 1 [file RHPB_A_2760023_SM8427.docx]

Table S1. Item-level confirmatory factor analysis results for the Arabic Asthma Numeracy Questionnaire (Ar-ANQ)*

| Item | Std. loading (λ) | SE | Item R² |
| --- | --- | --- | --- |
| ANQ1 | 0.80 |  | 0.64 |
| ANQ2 | 0.88 | 0.07 | 0.77 |
| ANQ3 | 0.93 | 0.07 | 0.86 |
| ANQ4 | 0.83 | 0.07 | 0.69 |

Standardized factor loadings, standard errors (SEs), and item R² are reported for the one-factor model.

Note. Item R² values were computed as λ². Model fit: χ²(2) = 3.62, p = 0.16; CFI = 0.983; TLI = 0.949; RMSEA = 0.045 (90% CI 0.00–0.12); SRMR = 0.022. Reliability reported in the validation study: Cronbach’s α = 0.80; composite reliability = 0.92; average variance extracted = 0.502.

* Reproduced from the previously published adult validation study conducted in the same cohort. Ref: Al-Qerem W, Jarab A, Al Oweidat K, Eberhardt J, Kasasbeh M, Al-sa’di L. Adaptation and validation for the Arabic version of asthma numeracy questionnaire (Ar-ANQ) in adults: a factor and Rasch analyses study. Libyan Journal of Medicine 2025;20:2538333.

# Table S2. Confirmatory factor analysis of the EQ-5D-3L items

| Item | Loading | SE | z | p | Standardized loading (λ) | R² |
| --- | --- | --- | --- | --- | --- | --- |
| mobility | 1.00 | 0.00 |  |  | 0.89 | 0.78 |
| selfcare | 5.40 | 13.92 | 0.39 | 0.698 | 1.00 | 0.99 |
| activity | 0.87 | 0.13 | 6.97 | < .001 | 0.86 | 0.73 |
| pain | 1.00 | 0.18 | 5.48 | < .001 | 0.88 | 0.78 |
| anxiety | 0.87 | 0.15 | 5.89 | < .001 | 0.86 | 0.74 |
| Unstandardized factor loadings, standard errors (SEs), z values, p values, standardized loadings (λ), and item R² are shown for the one-factor model estimated using DWLS/WLSMV for categorical indicators. **Note.** Model fit: χ²(5) = 19.54, p = 0.002; CFI = 0.997; TLI = 0.994; RMSEA = 0.086 (90% CI 0.048–0.127); SRMR = 0.054. Reliability: McDonald’s ω = 0.95; polychoric Cronbach’s α = 0.95. | | | | | | |

# Table S3. Confirmatory factor analysis of the MARS-5 adherence items.

| Item | Loading | SE | z | p | Standardized loading (λ) | R² |
| --- | --- | --- | --- | --- | --- | --- |
| Item1 | 1.00 | 0.00 |  |  | 0.59 | 0.35 |
| Item2 | 0.88 | 0.22 | 3.91 | < .001 | 0.54 | 0.29 |
| Item3 | 1.17 | 0.32 | 3.66 | < .001 | 0.65 | 0.42 |
| Item4 | 0.76 | 0.19 | 4.06 | < .001 | 0.49 | 0.24 |
| Item5 | 0.77 | 0.19 | 4.09 | < .001 | 0.49 | 0.24 |
| Unstandardized factor loadings, standard errors (SEs), z values, p values, standardized loadings (λ), and item R² are shown for the one-factor model estimated using DWLS/WLSMV for categorical indicators. **Note.** Model fit: χ²(5) = 2.86, p = 0.722; CFI = 1.000; TLI = 1.020; RMSEA = 0.000 (90% CI 0.000–0.051); SRMR = 0.028. Reliability: McDonald’s ω = 0.68; polychoric Cronbach’s α = 0.68. | | | | | | |
|  | | | | | | |

# Table S4. Confirmatory factor analysis of the asthma symptom control items.

| Item | Loading | SE | | z | p |  | Standardized loading (λ) | R² |
| --- | --- | --- | --- | --- | --- | --- | --- | --- |
| C1 | 1.00 | 0.00 | |  |  |  | 0.80 | 0.63 |
| C2 | 2.02 | 0.98 | | 2.07 | 0.039 |  | 0.94 | 0.88 |
| C3 | 0.93 | 0.20 | | 4.79 | < .001 |  | 0.77 | 0.60 |
| C4 | 1.22 | 0.37 | | 3.26 | 0.001 |  | 0.85 | 0.72 |
|  | | | Unstandardized factor loadings, standard errors (SEs), z values, p values, standardized loadings (λ), and item R² are shown for the one-factor model estimated using DWLS/WLSMV for categorical indicators. **Note.** Model fit: χ²(2) = 21.68, p < 0.001; CFI = 0.979; TLI = 0.938; RMSEA = 0.157 (90% CI 0.102–0.220); SRMR = 0.091. Reliability: McDonald’s ω = 0.91; polychoric Cronbach’s α = 0.88. | | | | | |

Table S5. Frequency of use of medication classes included in the primary regression analysis (N = 396)

| Medication | Frequency (%) |
| --- | --- |
| Montelukast | 88 (22.2%) |
| Inhaled corticosteroids | 306 (77.3%) |
| Antihistamines | 155 (39.1%) |
| Oral corticosteroids | 86 (21.7%) |
| Ipratropium bromide | 61 (15.4%) |
| Antibiotics | 65 (16.4%) |

Note. Percentages were calculated using the analytic sample of 396 participants. Participants could report more than one medication; therefore, categories were not mutually exclusive.
